# Supplementary material for: Inter- and Intra-Molecular Organocatalysis of SN2 Fluorination by Crown Ether: Kinetics and Quantum Chemical Analysis
Source: Molecules. 2021 May 15;26(10):2947. doi: 10.3390/molecules26102947 (PMC8156096; doi:10.3390/molecules26102947)
Supplement: Supplementary file 1 [file molecules-26-02947-s001.zip › molecules-1208751-SI.pdf]

Supporting Information

Inter- and Intramolecular Organocatalysis of S_N2 Fluorination by Crown Ethers: Kinetics and Quantum Chemical Analysis

Young-Ho Oh[1,†], Wonhyuck Yun[2,†], Chul-Hee Kim[2], Sung-Woo Jang[1], Sung-Sik Lee[1], Sungyul Lee[1,*] and Dong-Wook Kim[2,*]

[1] Department of Applied Chemistry, Kyung Hee University, Duckyoung-daero 1732, Yongin City, Gyeonggi 446-701, Republic of Korea.

[2] Department of Chemistry, Inha University, 100 Inha-ro, Nam-gu, Incheon 402-751, Republic of Korea.

† These authors contributed equally to this work.

General Remarks

Unless otherwise noted, all reagents and solvents were commercially available. TLC analysis was performed using Merck silica gel $60F_{254}$ plates. Visualization on TLC was monitored by UV light (254 nm). Flash chromatography was performed with 230-400 mesh silica gel. ^1H and ^{13}C NMR spectra were recorded on a 400 MHz spectrometer, and chemical shifts were reported in δ units (ppm) relative to tetramethylsilane. High resolution mass spectra were obtained at the Korea Basic Science Institute (Daegu, South Korea).

4-(3-Methanesulfonyloxypropyl)benzo-18-crown 6-ether (1). Triethylamine (111 mg, 1.10 mmol) and methanesulfonyl chloride (126 mg, 1.10 mmol) were added dropwise to a well-stirred solution of 4-(1-propanol)benzo-18-crown 6-ether (271 mg, 0.73 mmol) in CH_2Cl_2 (5 mL) at 0 °C. The mixture was stirred at 25 °C for overnight. After stirring, the reaction was quenched with water (5 mL) and extracted from the aqueous phase with MC (5 mL x 3). The organic layer was dried over sodium sulfate and evaporated under reduced pressure. The residue was purified by flash column chromatography (10% MeOH/MC) to yield 292 mg (0.65 mmol, 89%) of 4-(3-methanesulfonyloxypropyl)benzo-18-crown 6-ether as a yellow oil: ^1H NMR (400 MHz, $CDCl_3$) δ 1.99-2.06 (m, 2H), 2.66 (t, *J* = 7.4 Hz, 2H), 2.98 (s, 3H), 3.67-3.77 (m, 12H), 3.89-3.92 (m, 4H), 4.13-4.17 (m, 4H), 4.19 (t, *J* = 6.3 Hz, 2H), 6.68-6.71 (m, 2H), 6.81 (d, *J* = 8.0 Hz, 1H); ^{13}C NMR(100 MHz, $CDCl_3$) δ 30.86, 31.10, 37.45, 69.21, 69.34, 69.79, 70.81, 70.86, 114.63, 114.82, 121.16, 133.62, 147.56, 149.13 ; MS (FAB) 448 (M^+), 149 (100); HRMS (FAB) calcd for $C_{20}H_{32}O_9S$ (M^+) 448.1767, found 448.1769.

4-(3-Methanesulfonyloxypropyl)-1,2-dimethoxybenzene (2). Triethylamine (387 mg, 3.82 mmol) and methanesulfonyl chloride (350 mg, 3.06 mmol) were added dropwise to a well-stirred solution of 3-(3,4-Dimethoxyphenyl)-1-propanol (500mg, 2.55 mmol) in CH_2Cl_2 (10 mL) at 0 °C. The mixture was stirred at 25 °C for 3 h. After stirring, the reaction was quenched with water (10 mL) and extracted from the aqueous phase with MC (10 mL x 3). The organic layer was dried over sodium sulfate and evaporated under reduced pressure. The residue was purified by flash column chromatography (80% EtOAc/hexane) to yield 690mg (2.52 mmol, 99%) of 4-(3-methanesulfonyloxypropyl)-1,2-dimethoxybenzene as a colorless oil: ^1H NMR (400 MHz, $CDCl_3$) δ 1.99-2.06 (m, 2H), 2.67 (t, *J* = 7.6 Hz, 2H), 2.97 (s, 3H), 3.83 (s, 3H), 3.85 (s, 3H), 4.20 (t, *J* = 6.3 Hz, 2H), 6.70-6.71 (m, 2H), 6.78 (d, *J* = 8.5 Hz, 1H); ^{13}C NMR(100 MHz, $CDCl_3$) δ 30.84, 31.08, 37.32, 55.88, 55.94, 69.22, 111.42, 111.84, 120.33, 132.88, 147.52, 149.00; MS (EI) 274 (M^+), 151 (100); HRMS (EI) calcd for $C_{12}H_{18}O_5S$ (M^+) 274.0875, found 274.0878.

4-(3-Bromopropyl)-1,2-dimethoxybenzene (3). Triphenylphosphine (802 mg, 3.06 mmol) and N-bromosuccinimide (544 mg, 3.06 mmol) were added to a solution of 3-(3,4-Dimethoxyphenyl)-1-propanol (500 mg, 2.55 mmol) in CH_2Cl_2 (10 mL) at 0 °C under nitrogen atmosphere. The reaction mixture was stirred at 0 °C for 1 h. The reaction was quenched with saturated aqueous solution of $NaHCO_3$ (10 mL) and extracted from the aqueous phase with MC (10 mL x 3). The organic layer was dried over sodium sulfate and

evaporated under reduced pressure. The residue was purified by flash column chromatography (40% EtOAc/hexane) to yield 655 mg (2.53 mmol, 99%) of 4-(3-bromopropyl)-1,2-dimethoxybenzene as a colorless oil: ^{1}H NMR (400 MHz, $CDCl_3$) δ 2.06-2.13 (m, 2H), 2.68 (t, J = 7.3 Hz, 2H), 3.34 (t, J = 6.5 Hz, 2H), 3.81 (s, 3H), 3.83 (s, 3H), 6.69-6.71 (m, 2H), 6.76 (d, J = 8.5 Hz, 1H); ^{13}C NMR(100 MHz, $CDCl_3$) δ 33.03, 33.36, 34.17, 55.69, 55.76, 111.23, 111.74, 120.27, 132.93, 147.29, 148.78; MS (EI) 258 (M^+), 260 (M^+), 151 (100); HRMS (EI) calcd for $C_{11}H_{15}{}^{79}BrO_2$ (M^+) 258.0255, found 258.0254.

Typical Procedure for S_N2 Fluorination. CsF (46 mg, 0.3 mmol) was added to the mixture of substrates **1-3** (0.1 mmol) in CH_3CN (1.0 mL). The reaction mixture was stirred at 80 °C. At each time point, 0.1 mL of mixture was sampled. The reaction mixture was cooled to room temperature, solvents were removed under vacuum to concentrate the product. Ratios of compounds in reaction mixtures were determined using ^{1}H NMR spectroscopy.

4-Fluoropropylbenzo-18-crown 6-ether (1a). According to the typical procedure for fluorination, **1a** (37 mg, 99%) was obtained as a yellow oil after flash column chromatography (10% MeOH/MC); ^{1}H NMR (400 MHz, $CDCl_3$) δ 1.90-2.03 (m, 2H), 2.66 (t, J = 7.7 Hz, 2H), 3.68-3.77 (m, 12H), 3.90-3.93 (m, 4H), 4.12-4.16 (m, 4H), 4.45 (dt, J = 47.3, 6.0 Hz, 2H), 6.70-6.72 (m, 2H), 6.81 (d, J = 8.0 Hz, 1H); ^{13}C NMR(100 MHz, $CDCl_3$) δ 30.94 (d, J = 4.8 Hz), 32.27 (d, J = 19.3 Hz), 69.32, 69.49, 69.88, 69.90, 70.90, 70.94, 83.21 (d, J = 164.7 Hz), 114.74, 115.00, 121.22, 134.55, 147.47, 149.15; MS (FAB) 372 (M^+), 149 (100); HRMS (FAB) calcd for $C_{19}H_{29}FO_6$ (M^+) 372.1948, found 372.1950.

4-(3-Fluoropropyl)-1,2-dimethoxybenzene (2a). Prepared according to the typical procedure for fluorination except the use of 18-crown-6 (26 mg, 0.1 mmol), **2a** (19 mg, 98%) was obtained as a colorless oil after flash column chromatography (20% EtOAc/hexane); ^{1}H NMR (400 MHz, $CDCl_3$) δ 1.92-2.05 (m, 2H), 2.69 (t, J = 7.7 Hz, 2H), 3.85 (s, 3H), 3.87 (s, 3H), 4.47 (dt, J = 47.1, 6.0 Hz, 2H), 6.72-6.74 (m, 2H), 6.80 (d, J = 8.2 Hz, 1H); ^{13}C NMR(100 MHz, $CDCl_3$) δ 30.97 (d, J = 4.8 Hz), 32.29 (d, J = 19.3 Hz), 55.88, 55.98, 83.15 (d, J = 163.8 Hz), 111.39, 111.90, 120.37, 133.78, 147.42, 148.98; MS (EI) 198 (M^+), 151 (100); HRMS (EI) calcd for $C_{11}H_{15}FO_2$ (M^+) 198.1056, found 198.1057.

$^{1.}$ H and ^{13}C NMR spectra of Figure 1.

4-(3-Methanesulfonyloxypropyl)benzo-18-crown 6-ether (1)

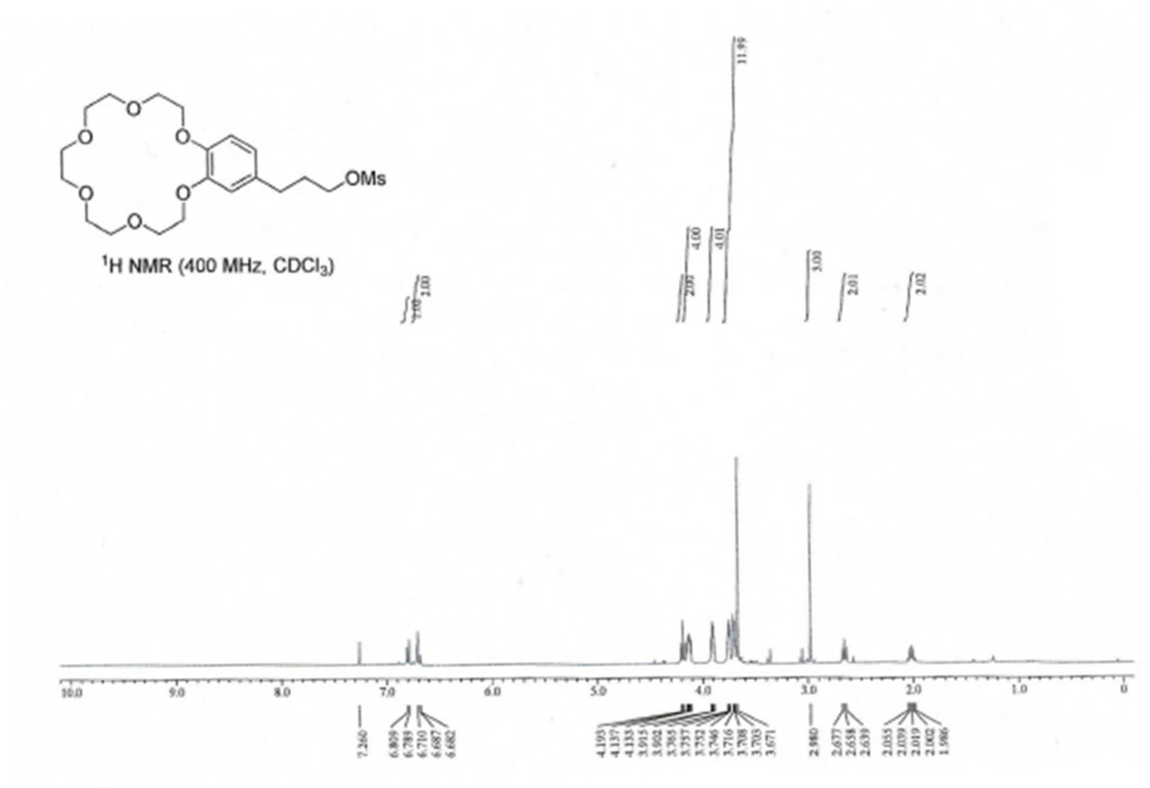

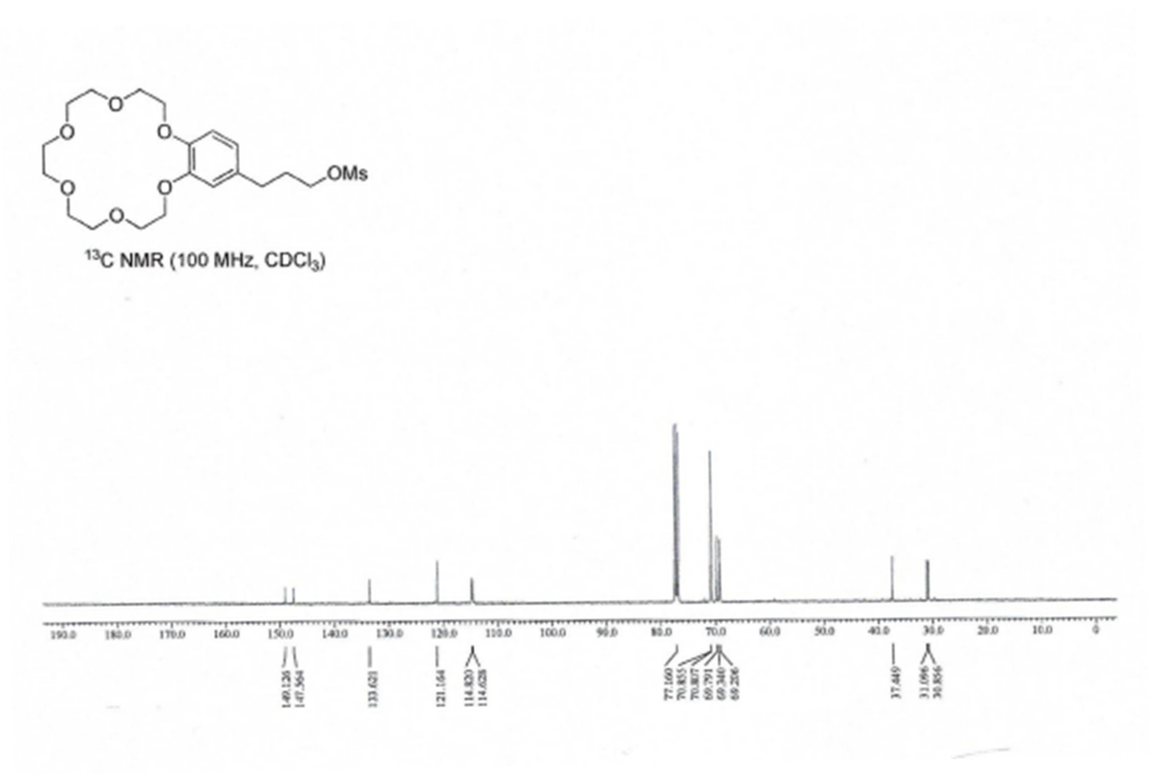

4. -(3-Methanesulfonyloxypropyl)-1,2-dimethoxybenzene (2)

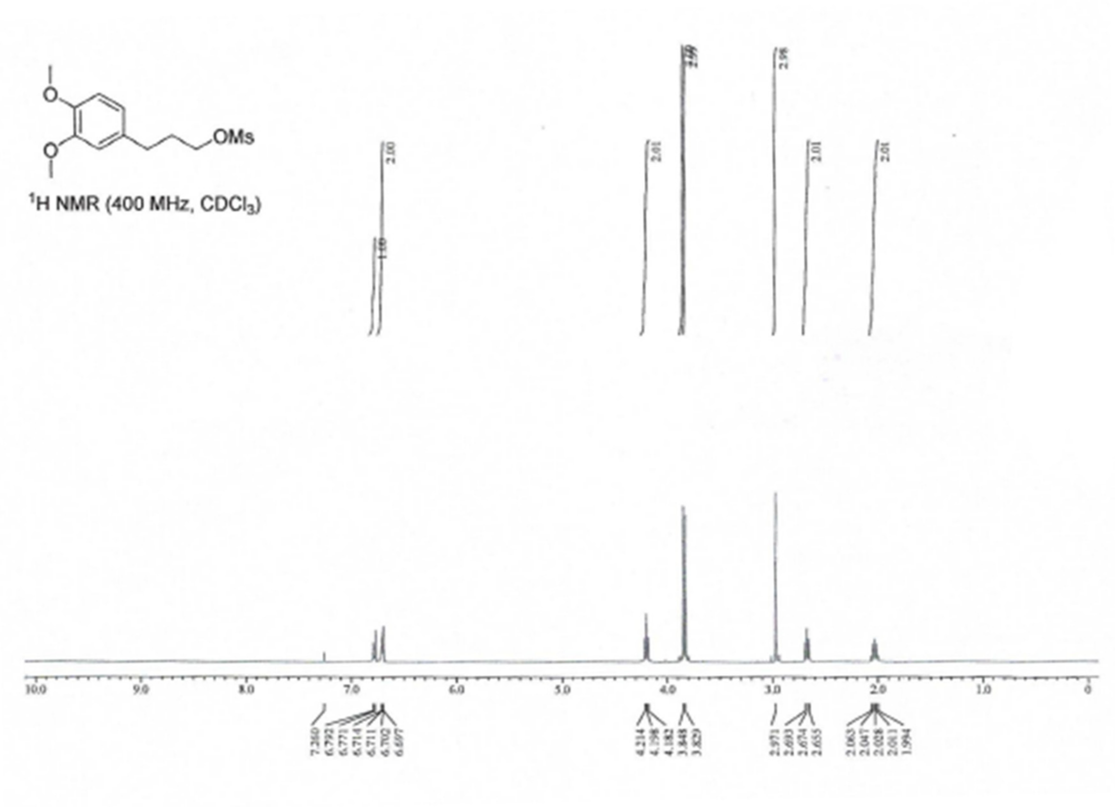

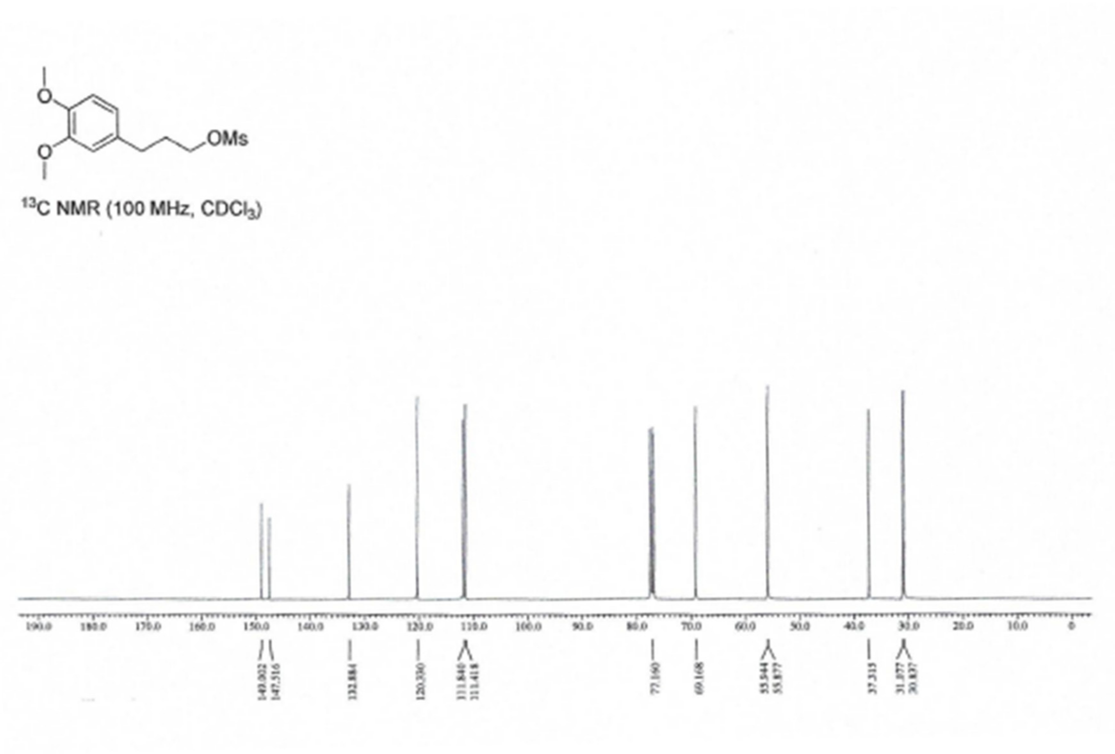

4-Fluoropropylbenzo-18-crown 6-ether (1a)

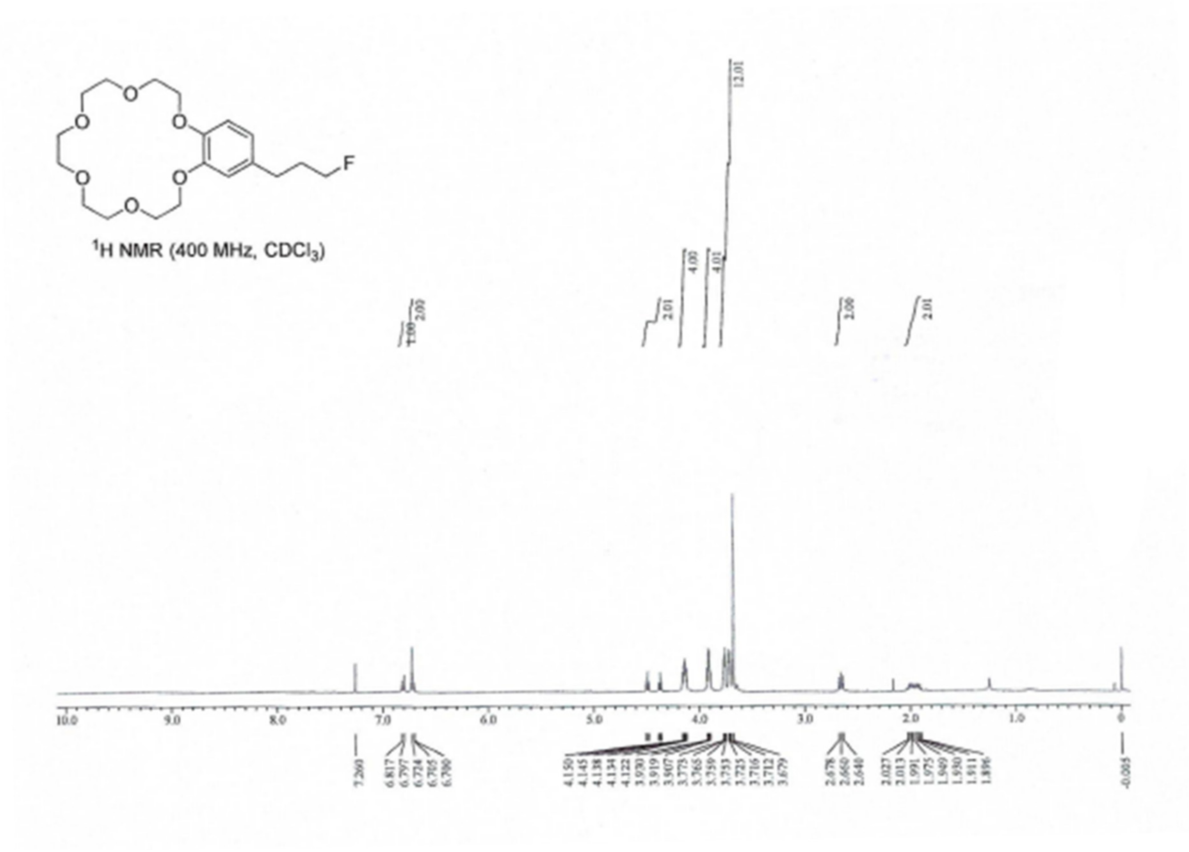

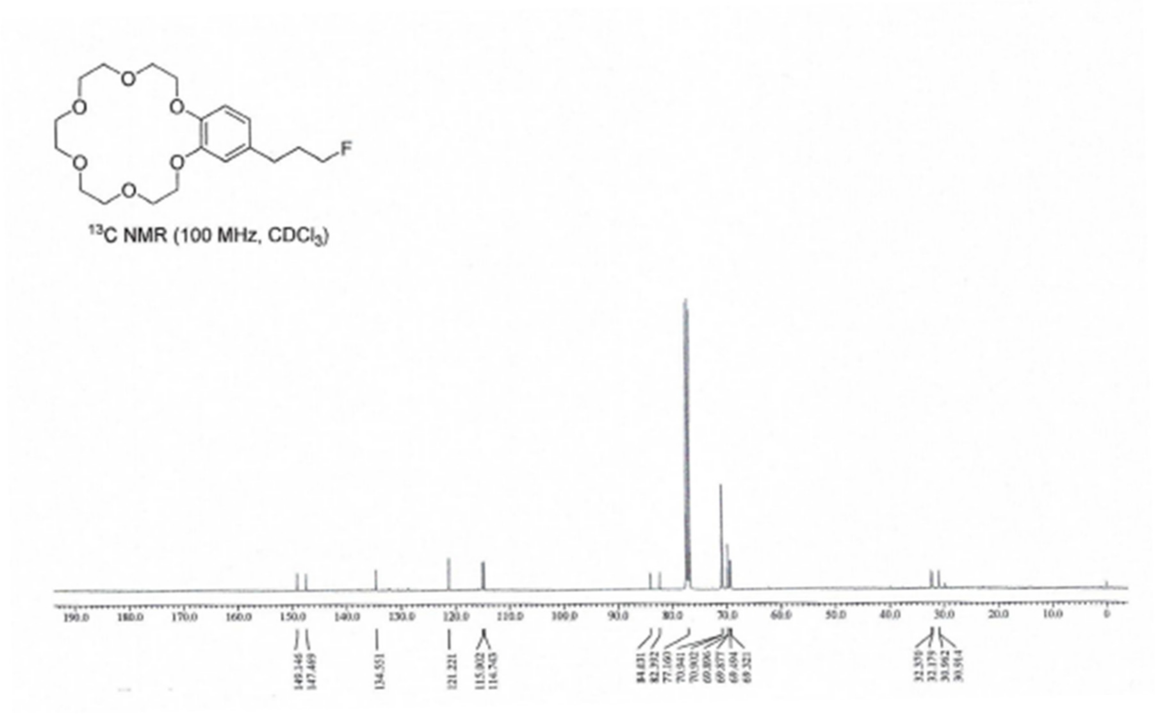

4. -(3-Fluoropropyl)-1,2-dimethoxybenzene (2a)

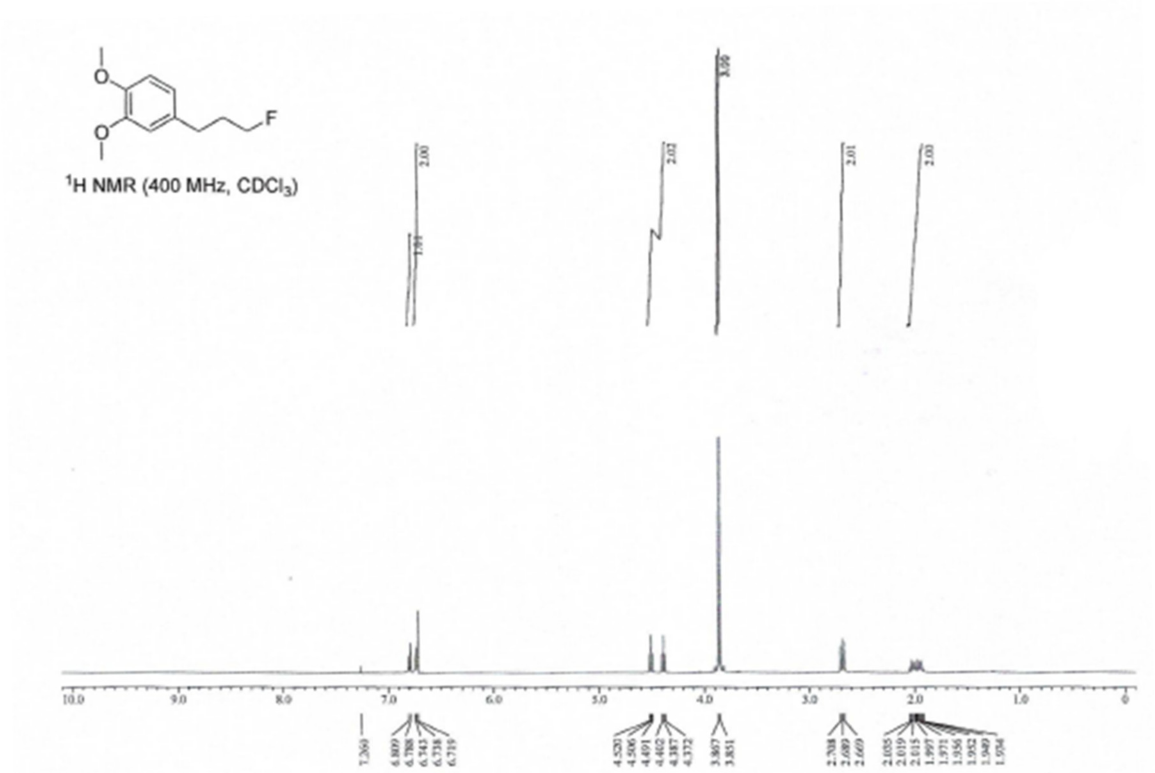

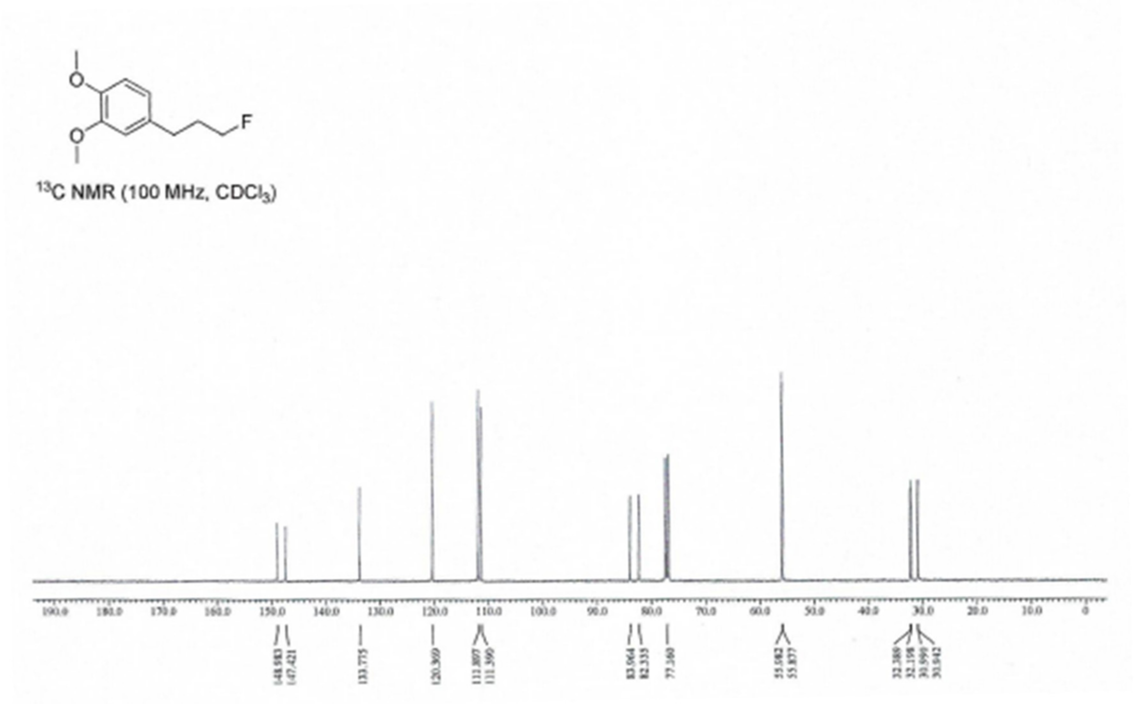

Structures and Cartesian coordinates

Figure 1S. Sub(1-OMs) - I

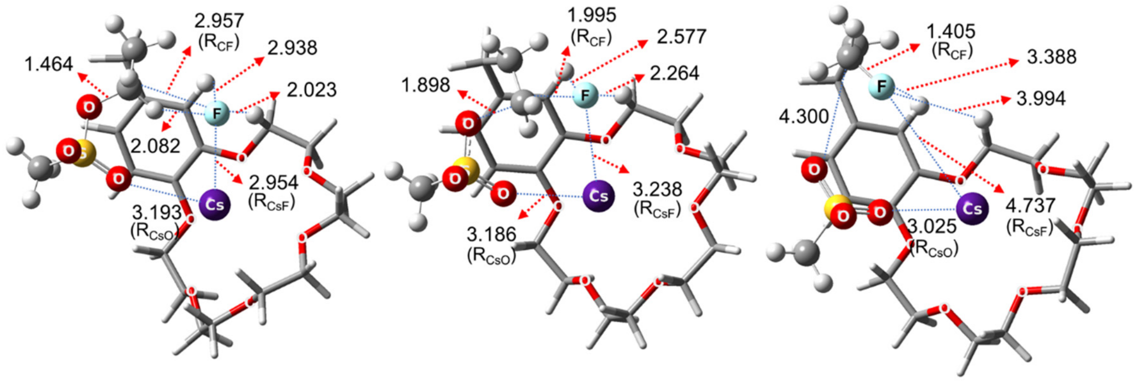

$G_{80℃}$ = 0.00 (pre-reaction complex) $G_{80℃}$ = 17.78 (Transition state) $G_{80℃}$ = -22.64 (post-reaction complex)

Sub(1-OMs) - I: pre-reaction complex

O	-1.76966800	-2.43387500	1.48873800
O	-2.38110800	2.71340200	0.00781200
O	-3.84338300	-1.77168800	-0.35599100
O	-0.01382500	-0.50441200	2.61595800
O	-3.90990600	0.79800300	-1.16243000
O	-0.14905400	1.93797500	1.70998400
C	-2.91418200	-3.23311800	1.25427000
C	-1.37733900	-2.43817600	2.84777800
C	-3.78582400	2.85493000	0.01673900
C	-1.74397600	3.53068500	0.97008400
C	-3.32973800	-3.07581000	-0.18472300
C	0.02119700	-1.89128300	2.93903000
C	-4.34927500	2.14277000	-1.18813500
C	-0.28099800	3.18118400	1.00661500
C	-4.19545100	-1.48671300	-1.69340300
C	1.17072900	0.07571300	2.27397800
C	-4.82513500	-0.11631000	-1.72881800
C	1.08788500	1.37952800	1.73477600
C	2.41016600	-0.53844000	2.37674800
C	2.22978700	1.98191600	1.22891600
C	3.54536300	0.07489600	1.83997200
C	3.46004900	1.31363300	1.22607800
H	-3.73951300	-2.92253300	1.90809700
H	-2.68594800	-4.28698900	1.45953500
H	-1.36939900	-3.46340100	3.23993100
H	-2.07184100	-1.84019400	3.45137900
H	-4.06976500	3.91530200	-0.02480000
H	-4.20021100	2.42082500	0.93684500
H	-2.19504900	3.38198100	1.96044500
H	-1.85421700	4.58873300	0.69786800
H	-2.46300300	-3.23929600	-0.84195400
H	-4.09296100	-3.82606300	-0.43036100
H	0.42347600	-2.03510700	3.94722800
H	0.65168200	-2.42843200	2.22273200
H	-5.44377500	2.19702000	-1.14655400
H	-4.01109900	2.62718500	-2.11274100
H	0.10662100	3.05020600	-0.01292700

H	0.27014200	3.96362500	1.53923800
H	2.50016300	-1.51919500	2.82502100
H	2.15721400	2.95045400	0.75178400
H	4.49417200	-0.45048900	1.86597100
C	4.61879600	1.87731800	0.44167100
H	4.94225100	2.83894800	0.85160000
H	5.46207700	1.18887900	0.52692300
C	4.27530600	2.07672400	-1.04767800
H	3.65807700	2.97121000	-1.17883700
H	5.19977000	2.23631700	-1.60829300
C	3.47001300	0.94690400	-1.65816000
H	2.44212400	0.94987600	-1.29163000
H	3.45303700	1.02672000	-2.74769600
O	4.12236900	-0.31569400	-1.30630000
S	3.26543800	-1.64160700	-1.53275800
O	1.92403900	-1.46111100	-0.99876200
O	3.37438800	-2.04751300	-2.92309000
C	4.18504500	-2.74116900	-0.50573600
H	3.73029600	-3.72526800	-0.61989100
H	5.21771900	-2.75299000	-0.85043500
H	4.10946000	-2.39264100	0.52250200
F	0.74343400	2.09111900	-1.67654000
Cs	-0.93438200	-0.09237500	-0.60733800
H	-3.30377800	-1.51473300	-2.33669400
H	-4.91179400	-2.22524600	-2.07684900
H	-5.06018500	0.14795400	-2.76827600
H	-5.75795200	-0.11820900	-1.14992900

Electronic Energy (Hartree)	Zero point correction (kcal/mol)	Electronic and Zero point correction (Hartree)	Gibbs free Energy (kcal/mol)	Electronic and Gibbs Free Energy (Hartree)	Number of Imaginary frequencies
-1976.071956	332.2298847	-1975.5425	278.2075918	-1975.628604	0

Sub(1-OMs) - I: transition state

O	-1.76473200	-2.47329400	1.52332800
O	-2.40286300	2.45769000	0.11559500
O	-3.72448800	-1.61575600	-0.37423800
O	-0.03087600	-0.65055000	2.82073800
O	-3.53324300	0.80771200	-1.88664400
O	-0.33609600	1.79744100	2.02160300
C	-2.97795700	-3.15762700	1.27754800
C	-1.33857700	-2.62243500	2.86393100
C	-3.71089200	2.70073300	-0.36811100
C	-2.03916100	3.25458900	1.22641900
C	-3.37151400	-2.96871500	-0.16244900
C	0.05185700	-2.06291400	2.98010900
C	-3.76968500	2.20308800	-1.79615000
C	-0.55883900	3.08770500	1.44599000
C	-4.32636300	-1.41559800	-1.63910600
C	1.10730200	-0.00134800	2.46035500
C	-4.71285500	0.03282200	-1.76773900

C	0.93898500	1.32699200	2.00787200
C	2.37356800	-0.56721300	2.45303800
C	2.02576300	2.01646600	1.49848400
C	3.44891600	0.12582200	1.88617900
C	3.27586500	1.39690000	1.36607700
H	-3.77204100	-2.77314000	1.93099700
H	-2.85193200	-4.22980700	1.47781800
H	-1.31324600	-3.68473100	3.13943300
H	-2.02404000	-2.10338000	3.54617500
H	-3.92674500	3.77709700	-0.36211600
H	-4.45389600	2.19751400	0.26448300
H	-2.60501000	2.96523100	2.12122500
H	-2.24132800	4.31182500	1.01337400
H	-2.53762000	-3.25424500	-0.81970600
H	-4.22421300	-3.62292900	-0.38575100
H	0.48746600	-2.31173500	3.95318700
H	0.67230500	-2.49591200	2.18835800
H	-4.73655800	2.46175600	-2.24414700
H	-2.98002600	2.69425400	-2.36980200
H	-0.04969800	3.15056300	0.47839100
H	-0.18054900	3.86188500	2.12156100
H	2.52460000	-1.57044500	2.83030800
H	1.88412600	2.99691600	1.06618900
H	4.41398400	-0.36283700	1.80658200
C	4.30792400	2.01616200	0.45716800
H	4.67907100	2.96613200	0.85299200
H	5.15690100	1.33516300	0.37864800
C	3.72058200	2.27386300	-0.94904200
H	3.16786000	3.21319800	-0.96333100
H	4.52940900	2.35956700	-1.67766800
C	2.73737900	1.24435300	-1.42198200
H	2.16901800	0.66375500	-0.72434800
H	2.48934400	1.19258500	-2.46807900
O	3.98284300	-0.18002100	-1.56521900
S	3.37773000	-1.56385900	-1.75459800
O	1.96194200	-1.58527700	-1.34847000
O	3.63747800	-2.08160300	-3.09893900
C	4.26742900	-2.58278100	-0.60362000
H	3.89124200	-3.60063600	-0.70283400
H	5.32848400	-2.54255300	-0.84595800
H	4.08429200	-2.20414100	0.40033200
F	1.10465700	2.38475800	-1.31279400
Cs	-0.78360400	-0.14937700	-0.60634900
H	-3.62948700	-1.68971600	-2.44388800
H	-5.22392900	-2.04048500	-1.73297000
H	-5.34107000	0.16680600	-2.65791200
H	-5.29217900	0.33118400	-0.88549600

Electronic Energy (Hartree)	Zero point correction (kcal/mol)	Electronic and Zero point correction (Hartree)	Gibbs free Energy (kcal/mol)	Electronic and Gibbs Free Energy (Hartree)	Number of Imaginary frequencies
-1976.042369	331.1198204	-1975.5147	277.425715	-1975.600263	1

Sub(1-OMs) - I: post-reaction complex

O	1.91127900	2.08901100	1.61996800
O	1.88594800	-3.08130000	-0.16824100
O	3.87098400	1.18728800	-0.10180300
O	-0.32688200	0.35175500	2.15247300
O	4.09973900	-1.40654500	-0.83235900
O	-0.26362600	-2.14484700	1.42886300
C	3.27913700	2.27437900	1.93156000
C	1.10170600	2.13683300	2.77890400
C	3.17337300	-3.56113500	-0.51250100
C	1.27063200	-3.86147200	0.83773600
C	4.07848900	2.36477100	0.65621500
C	-0.30908000	1.75327200	2.42369100
C	3.79439400	-2.61178600	-1.50386600
C	-0.18114400	-3.47655500	0.92848900
C	4.99576600	0.78482700	-0.85753200
C	-1.52228000	-0.19304800	1.79903100
C	4.59781500	-0.40509300	-1.69534700
C	-1.49158100	-1.55551000	1.41890700
C	-2.72879600	0.48608400	1.77804600
C	-2.66515100	-2.19170300	1.04687400
C	-3.90088600	-0.16395400	1.37636400
C	-3.88397900	-1.49832900	1.00813900
H	3.63851200	1.43045900	2.53539500
H	3.41992100	3.19886200	2.50805300
H	1.09949000	3.15220600	3.19649700
H	1.48869000	1.44999700	3.54310200
H	3.09513800	-4.56138000	-0.95730700
H	3.80832400	-3.62523300	0.38057600
H	1.77145600	-3.71005000	1.80256300
H	1.32621100	-4.92757600	0.58197800
H	3.77718600	3.24272700	0.07126200
H	5.13683400	2.47336800	0.92214300
H	-0.96637900	1.98194400	3.26929200
H	-0.64348300	2.29584200	1.53361300
H	4.70480200	-3.05934900	-1.92328500
H	3.09163600	-2.42527800	-2.32900000
H	-0.63308000	-3.53029900	-0.06803400
H	-0.70848300	-4.16440800	1.59779100
H	-2.77012000	1.53117100	2.05354600
H	-2.64776600	-3.23413300	0.75221300
H	-4.83008300	0.39472000	1.34414200
C	-5.12805800	-2.19266600	0.50935600
H	-5.32968300	-3.08062500	1.11678200
H	-5.98016100	-1.51838000	0.63248700
C	-5.04212700	-2.61785400	-0.96348300
H	-4.29180200	-3.40349700	-1.09731400
H	-6.00400600	-3.04196000	-1.26637300
C	-4.71157300	-1.48855900	-1.91469200
H	-5.23396900	-0.56468200	-1.65260800
H	-4.94432300	-1.75880600	-2.94609000

O	-3.35015800	2.57503000	-1.55697500
S	-2.01518600	3.13028300	-1.26322100
O	-1.03479500	2.09793600	-0.84932700
O	-1.49704200	4.01020100	-2.32887500
C	-2.23319700	4.18214700	0.16619300
H	-1.26952300	4.60943500	0.44278400
H	-2.93252700	4.97670600	-0.09251500
H	-2.63222600	3.58692700	0.98687100
F	-3.33545700	-1.20953600	-1.87532400
Cs	1.08977200	-0.04644100	-0.64819800
H	5.33775100	1.59210900	-1.51817800
H	5.81880400	0.51166500	-0.18457600
H	5.47391700	-0.76604100	-2.25022300
H	3.82922400	-0.11705700	-2.42755700

Electronic Energy (Hartree)	Zero point correction (kcal/mol)	Electronic and Zero point correction (Hartree)	Gibbs free Energy (kcal/mol)	Electronic and Gibbs Free Energy (Hartree)	Number of Imaginary frequencies
-1976.101764	331.3143483	-1975.5738	274.2693422	-1975.664688	0

Figure 2S. Sub(1-OMs) - II

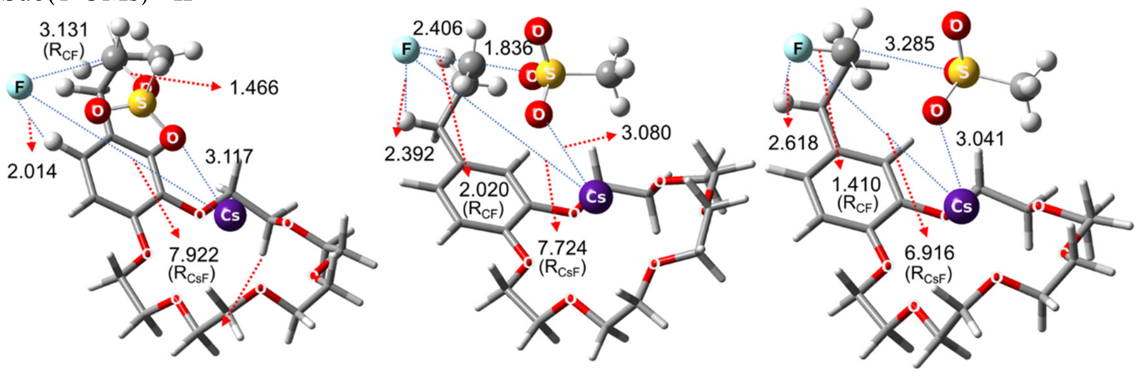

$G_{80℃}$ = 0.00 (pre-reaction complex)

$G_{80℃}$ = 17.46 (Transition state)

$G_{80℃}$ = -28.28 (post-reaction complex)

Sub(1-OMs) - II: pre-reaction complex

O	-2.24873000	-2.20065800	1.58708400
O	-2.14844000	2.88041300	-0.27294400
O	-4.00137900	-1.78413500	-0.65815200
O	-0.16305400	-0.39627900	2.28583800
O	-4.31767300	1.07374100	-0.68950600
O	-0.02915200	2.00882800	1.34759900
C	-3.58505200	-2.67051800	1.57332200
C	-1.74052600	-2.05239100	2.89895400
C	-3.48764300	3.29511900	-0.46606700
C	-1.45051900	3.74376900	0.60280200
C	-3.97165900	-2.95669400	0.13987800
C	-0.27674900	-1.71556400	2.80764800
C	-4.18993200	2.31167600	-1.36349300
C	-0.02322100	3.28034600	0.70907200

C	-5.25036200	-1.11537500	-0.62135800
C	1.09233500	0.05551600	2.00116100
C	-5.13758800	0.16625600	-1.40302700
C	1.16547800	1.36646800	1.47949300
C	2.25880300	-0.67536100	2.16857200
C	2.39726000	1.90355400	1.13612800
C	3.49397900	-0.13268100	1.79948300
C	3.57144400	1.15152400	1.28225300
H	-4.25477600	-1.92594600	2.02411100
H	-3.66439100	-3.59844600	2.15540000
H	-1.84971800	-2.99187300	3.45559100
H	-2.28126500	-1.26326300	3.43714300
H	-3.51273200	4.28925100	-0.93151200
H	-4.00894300	3.35117700	0.49852600
H	-1.92188900	3.74200100	1.59415400
H	-1.45998900	4.77108200	0.21605100
H	-3.22519700	-3.62133600	-0.30172000
H	-4.94388700	-3.46312600	0.11287800
H	0.18804000	-1.77108000	3.79776700
H	0.21616400	-2.43244200	2.14175800
H	-5.18113300	2.71003300	-1.61647300
H	-3.62451100	2.18104800	-2.29719700
H	0.42016500	3.19577800	-0.29003100
H	0.55480700	4.00150600	1.29623700
H	2.21817300	-1.68070600	2.56760900
H	2.45941700	2.90667300	0.72998400
H	4.41531100	-0.71270900	1.84787100
C	4.90179900	1.68564300	0.80883400
H	5.04570300	2.71632700	1.14598200
H	5.68079000	1.04230000	1.22628700
C	5.05538800	1.62950400	-0.71940500
H	4.36130100	2.31070800	-1.22180500
H	6.06773600	1.94853200	-0.98402300
C	4.85801100	0.22790100	-1.26908900
H	5.33693800	0.10814300	-2.24373700
H	5.22901900	-0.51248600	-0.55108100
O	3.41621000	0.05502200	-1.46726700
S	2.84707700	-1.38213800	-1.82739200
O	3.52182500	-2.40454200	-1.05470100
O	1.40661500	-1.24750700	-1.70406000
C	3.27306600	-1.57154400	-3.53451700
H	2.86578500	-2.52796200	-3.86201300
H	2.82819200	-0.74943900	-4.09283500
H	4.35881700	-1.57214500	-3.62616300
F	6.30426600	-1.01241900	1.21597900
Cs	-1.33471000	-0.15618000	-0.70021800
H	-6.02648300	-1.74809700	-1.07065200
H	-5.54256200	-0.88600200	0.41046700
H	-6.13871800	0.59385900	-1.54296400
H	-4.70840600	-0.03743800	-2.39411300

Electronic Energy (Hartree)	Zero point correction (kcal/mol)	Electronic and Zero point correction (Hartree)	Gibbs free Energy (kcal/mol)	Electronic and Gibbs Free Energy (Hartree)	Number of Imaginary frequencies
-1976.069009	331.9688407	-1975.54	276.6476032	-1975.628143	0

Sub(1-OMs) - II: transition state

O	3.01595600	2.63624300	-0.56523300
O	1.07678500	-1.93340500	1.82772100
O	4.37293100	0.22069300	-0.77165800
O	0.43832400	2.56776700	0.66322700
O	3.62440700	-2.03977800	0.53618900
O	-0.36373500	0.47954600	1.99541700
C	4.39506300	2.54590100	-0.25433500
C	2.41263100	3.73669100	0.08930900
C	2.05130000	-2.93618900	2.06431100
C	0.44091600	-1.50926100	3.01966400
C	5.03156200	1.44105800	-1.05869400
C	0.91997400	3.67593300	-0.09167400
C	2.82596600	-3.17728700	0.79431800
C	-0.78114800	-0.70789100	2.66267100
C	5.21848000	-0.91274100	-0.81149200
C	-0.90629300	2.34683000	0.66852700
C	4.36253400	-2.14703100	-0.66467200
C	-1.34240800	1.22933100	1.41503800
C	-1.84166700	3.11303800	-0.00886900
C	-2.69432200	0.94583000	1.49969600
C	-3.20216400	2.78792400	0.05630700
C	-3.64428100	1.71437300	0.81292600
H	4.51843900	2.33861000	0.81686200
H	4.90135900	3.49314700	-0.48406400
H	2.78547000	4.67955600	-0.33068100
H	2.65011200	3.71827200	1.16085900
H	1.56361300	-3.86550700	2.38416700
H	2.74020600	-2.61312200	2.85479600
H	1.13274100	-0.91122500	3.62586300
H	0.12036200	-2.37578300	3.61174300
H	4.96848100	1.65492000	-2.13290900
H	6.09042700	1.38614100	-0.77935900
H	0.47160800	4.60551500	0.27379000
H	0.66639100	3.55270700	-1.15111200
H	3.45374300	-4.06976100	0.91412800
H	2.13175800	-3.35787500	-0.04031800
H	-1.43188800	-1.29492700	2.00611300
H	-1.33627800	-0.45542100	3.57254800
H	-1.52718700	3.96223500	-0.60142700
H	-3.02880800	0.09498000	2.07654900
H	-3.91592900	3.38911700	-0.49671900
C	-5.10855500	1.34700700	0.88500600
H	-5.50847800	1.60803600	1.87058000
H	-5.65727200	1.91916200	0.13645500

C	-5.38080300	-0.13667000	0.61784500
H	-4.94046700	-0.76308600	1.39595400
H	-6.45666300	-0.30788400	0.62533900
C	-4.86628000	-0.57668400	-0.72543400
H	-5.32166600	-1.41082900	-1.22941900
H	-4.25952600	0.09238200	-1.31363400
O	-3.44349600	-1.57770300	-0.13777400
S	-2.42665800	-1.90758900	-1.22925900
O	-3.08102300	-2.51754300	-2.38855900
O	-1.57938600	-0.74710400	-1.53567200
C	-1.38979700	-3.13493400	-0.46796400
H	-0.71040500	-3.49832800	-1.23979300
H	-0.82801500	-2.68161500	0.34899100
H	-2.01807200	-3.94757300	-0.10697300
F	-6.32520000	0.47241800	-1.64724600
Cs	1.32016900	-0.11184400	-0.71540100
H	5.76127200	-0.96619200	-1.76401500
H	5.95078600	-0.86038400	0.00428400
H	3.68094900	-2.23943500	-1.52298200
H	5.00662700	-3.03588300	-0.64936100

Electronic Energy (Hartree)	Zero point correction (kcal/mol)	Electronic and Zero point correction (Hartree)	Gibbs free Energy (kcal/mol)	Electronic and Gibbs Free Energy (Hartree)	Number of Imaginary frequencies
-1976.040661	330.7546099	-1975.5136	276.3212983	-1975.600316	1

Sub(1-OMs) - II: post-reaction complex

O	2.87100900	2.63838700	-0.43259800
O	1.05424400	-1.97936100	1.76587000
O	4.27019600	0.28748700	-1.37777800
O	0.37964900	2.52403600	0.94380800
O	3.63033500	-1.72063800	0.58941200
O	-0.52196000	0.31301500	1.92753900
C	4.28482100	2.55772700	-0.48500700
C	2.42302800	3.62447700	0.47712100
C	2.14622600	-2.81938600	2.09015700
C	0.22841100	-1.71869300	2.88435900
C	4.67029600	1.62777000	-1.61363100
C	0.92332100	3.71756000	0.39136500
C	3.02798700	-2.96872800	0.87858600
C	-0.97664700	-0.93949900	2.43340300
C	5.22690300	-0.46718500	-0.65349300
C	-0.97120700	2.35999400	0.87899500
C	4.65700200	-1.83386500	-0.37921400
C	-1.46203200	1.14655500	1.41126900
C	-1.86119200	3.26767000	0.32671600
C	-2.81896500	0.87387600	1.36887100
C	-3.23091100	2.97766900	0.29089300
C	-3.72259400	1.79023800	0.80968200
H	4.67915200	2.20033800	0.47558400
H	4.71208600	3.55042100	-0.68106600

H	2.85020200	4.60164400	0.21726900
H	2.72817800	3.37315400	1.50112900
H	1.78453000	-3.80762700	2.40307900
H	2.72468100	-2.38258700	2.91443200
H	0.78851800	-1.15547000	3.64175600
H	-0.11452400	-2.65940800	3.33540300
H	4.16388800	1.95196600	-2.52610800
H	5.75242200	1.68074300	-1.78235400
H	0.56981700	4.58896100	0.95281200
H	0.62180800	3.82181100	-0.65693800
H	3.79747000	-3.72229700	1.08931100
H	2.43525600	-3.31503400	0.01890000
H	-1.52653900	-1.47809200	1.65593700
H	-1.64261300	-0.78032300	3.28945000
H	-1.50421800	4.20311600	-0.08410500
H	-3.18688100	-0.07879700	1.72821100
H	-3.91245400	3.69564500	-0.15279200
C	-5.19590000	1.45655100	0.75985200
H	-5.60000000	1.42176800	1.77673300
H	-5.72085600	2.25892300	0.23595800
C	-5.49212200	0.11695800	0.07222300
H	-5.04632100	-0.71275500	0.62769700
H	-6.57408100	-0.04251400	0.04213300
C	-4.93318500	0.04125400	-1.32723100
H	-5.21566700	-0.88774400	-1.82537000
H	-3.84579200	0.13767300	-1.34033600
O	-3.21311900	-2.29520600	0.21241400
S	-2.32290500	-2.37197500	-0.96680800
O	-2.99058200	-2.91916700	-2.16266900
O	-1.60864000	-1.09993800	-1.22856100
C	-1.03598000	-3.53717700	-0.53200500
H	-0.37136500	-3.65395300	-1.38846500
H	-0.48161300	-3.14273000	0.32098800
H	-1.49461400	-4.49308400	-0.28065800
F	-5.45391700	1.10143900	-2.09653200
Cs	1.22278600	-0.09931000	-0.75097200
H	6.14710000	-0.56927100	-1.24294600
H	5.47506000	0.02079400	0.29703400
H	4.26085700	-2.26314000	-1.31013100
H	5.45240000	-2.49296700	-0.00833900

Electronic Energy (Hartree)	Zero point correction (kcal/mol)	Electronic and Zero point correction (Hartree)	Gibbs free Energy (kcal/mol)	Electronic and Gibbs Free Energy (Hartree)	Number of Imaginary frequencies
-1976.112071	331.7680377	-1975.5834	275.3907017	-1975.673208	0

Figure 3S. Sub(2-OMs)

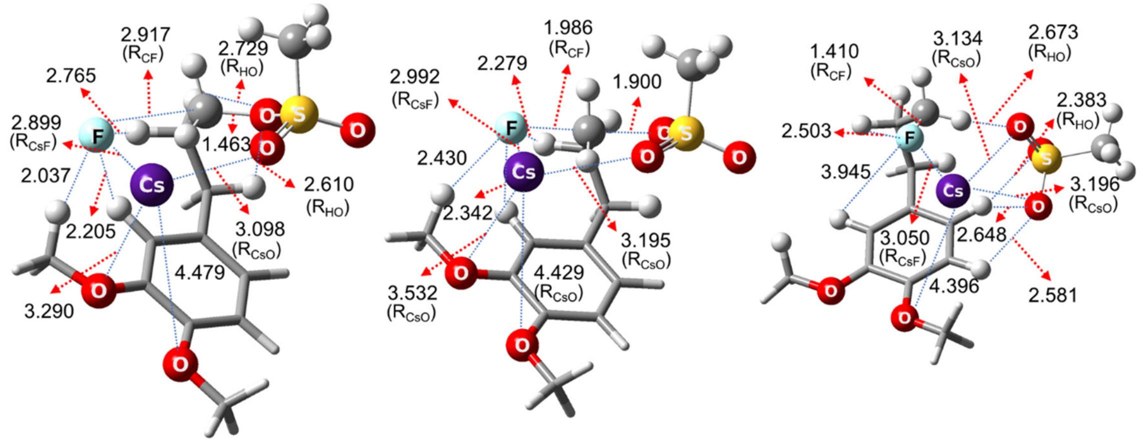

$G_{80℃}$ = 0.00 (pre-reaction complex) $G_{80℃}$ = 18.18 (Transition state) $G_{80℃}$ = -20.80 (post-reaction complex)

Sub(2-OMs): pre-reaction complex

O	-3.47037900	1.16670500	-1.28219700
O	-2.95651000	-0.23722300	0.83839500
C	-3.76469400	1.90746400	-2.45555700
C	-2.86267300	-0.85965300	2.12191700
C	-2.38208600	1.55052700	-0.56913500
C	-2.10579900	0.79464000	0.59248600
C	-1.53327400	2.59361500	-0.90919200
C	-1.00042000	1.09653300	1.37430300
C	-0.42610500	2.89426000	-0.10717300
C	-0.14840400	2.16044800	1.03677500
H	-2.94842800	1.84381500	-3.18192000
H	-3.96337600	2.95809900	-2.22237100
H	-3.07517400	-0.13096200	2.91144900
H	-1.87079900	-1.29717800	2.28308900
H	-1.72093900	3.18341300	-1.79712700
H	-0.74414700	0.43467200	2.19446000
H	0.22344500	3.71512100	-0.39304000
C	1.03470100	2.51711600	1.91127300
H	1.71310200	3.14398200	1.32821900
H	0.69157700	3.12796900	2.75314100
C	1.81824300	1.32941700	2.48218300
H	2.71237200	1.70852800	2.98378800
H	1.23194600	0.78897700	3.22998500
C	2.20315200	0.30219500	1.44216600
H	2.90755100	-0.42761600	1.84776400
H	1.32391100	-0.22094200	1.07315600
F	0.14679600	-1.57935300	2.30196500
Cs	-0.62676900	-2.18568700	-0.42574900
H	-3.63144500	-1.63127400	2.12827500
H	-4.66054000	1.45781400	-2.87955400
O	3.44716300	1.29304300	-1.97738100
S	2.98912000	0.27849300	-1.05568000
O	2.86350800	1.01519700	0.34890700
O	1.74778600	-0.42204100	-1.34654100

C	4.26244300	-0.91496400	-0.77509900
H	3.92182600	-1.61551800	-0.01272300
H	4.42983000	-1.43377800	-1.71906300
H	5.16141800	-0.38909700	-0.45728000

Electronic Energy (Hartree)	Zero point correction (kcal/mol)	Electronic and Zero point correction (Hartree)	Gibbs free Energy (kcal/mol)	Electronic and Gibbs Free Energy (Hartree)	Number of Imaginary frequencies
-1361.994961	187.7672	-1361.7	153.7367	-1361.764922	0

Sub(2-OMs) : transition state

O	-3.53565400	0.97371300	-1.42262800
O	-3.20763200	-0.23948000	0.85466900
C	-3.72370700	1.60305700	-2.68053600
C	-3.08442000	-0.86782900	2.12697300
C	-2.48789300	1.39677600	-0.67346900
C	-2.32717100	0.75848900	0.57880200
C	-1.57342600	2.36707300	-1.05425500
C	-1.28729700	1.13025800	1.41325800
C	-0.50487400	2.70681400	-0.21298700
C	-0.35206500	2.10162100	1.02376900
H	-2.86880300	1.43147600	-3.34185200
H	-3.88595000	2.67893200	-2.56420900
H	-3.29683000	-0.15820900	2.93271000
H	-2.08237800	-1.28438500	2.27175200
H	-1.66908500	2.85917900	-2.01356700
H	-1.13342000	0.60019300	2.34193500
H	0.21535700	3.44615600	-0.54772800
C	0.82207200	2.41531000	1.92440000
H	1.56606000	2.96608700	1.34693500
H	0.50226800	3.06535100	2.74531500
C	1.47724500	1.16537900	2.53487000
H	2.43614600	1.43624200	2.98115400
H	0.85677200	0.74623900	3.32709400
C	1.68343400	0.05898100	1.54763500
H	2.37705500	-0.73883700	1.75224300
H	1.05101500	0.00432200	0.68683500
F	0.34692200	-1.21354200	2.28153100
Cs	-0.50377800	-2.10393400	-0.44557100
H	-3.82649600	-1.66376900	2.13859600
H	-4.61257800	1.14929100	-3.11446600
O	3.68105600	1.42720100	-1.81218200
S	3.18551100	0.40298400	-0.89678400
O	2.95679000	0.98972800	0.48829800
O	1.99698300	-0.33593600	-1.35535200
C	4.47333700	-0.79795300	-0.66104300
H	4.12621300	-1.53527500	0.06298200
H	4.67645500	-1.27435200	-1.61961600
H	5.36179200	-0.28784500	-0.29187900

Electronic Energy (Hartree)	Zero point correction (kcal/mol)	Electronic and Zero point correction (Hartree)	Gibbs free Energy (kcal/mol)	Electronic and Gibbs Free Energy (Hartree)	Number of Imaginary frequencies
-1361.962819	186.3816	-1361.67	151.8215	-1361.735944	1

Sub(2-OMs) : post-reaction complex

O	2.76498900	-2.06307500	1.21116200
O	4.38099200	-0.36049500	0.11754600
C	1.89228900	-3.01797300	1.79232200
C	5.29445600	0.54210200	-0.48520100
C	2.28772200	-0.79933700	1.07803300
C	3.16997900	0.13111200	0.47711800
C	1.02869100	-0.37723100	1.47577800
C	2.76197600	1.44441100	0.29325900
C	0.63192400	0.95219100	1.28638600
C	1.48580500	1.86943800	0.69817100
H	0.97926300	-3.13396000	1.19926700
H	1.62583000	-2.74204500	2.81742700
H	5.54274600	1.36558800	0.19127100
H	4.89497400	0.94584400	-1.42061700
H	0.31940800	-1.06866600	1.91356300
H	3.43306600	2.16031600	-0.16706000
H	-0.36863800	1.24804400	1.58429600
C	1.03552500	3.28511500	0.43275600
H	0.22582900	3.53431000	1.12476100
H	1.85669400	3.98068000	0.62791300
C	0.52985200	3.50760800	-1.00079600
H	0.16796400	4.53634200	-1.09200500
H	1.34456400	3.38995400	-1.72285800
C	-0.59757100	2.58168500	-1.40247000
H	-1.13061300	2.95863500	-2.27708300
H	-1.30416600	2.39851700	-0.58901300
F	-0.07178800	1.32701800	-1.77494000
Cs	-0.80491900	-1.59721400	-1.31185400
H	6.19219900	-0.03568900	-0.69646400
H	2.43964900	-3.95855800	1.80228800
O	-2.18271300	-0.67323400	1.41940300
S	-3.03718000	0.42095600	0.90006600
O	-2.69682600	1.74297000	1.46150900
O	-3.09422500	0.41334400	-0.57997500
C	-4.69324100	0.06691300	1.46522200
H	-5.36229500	0.83841700	1.08590800
H	-4.99212500	-0.91074200	1.08869800
H	-4.69501700	0.06906700	2.55449200

Electronic Energy (Hartree)	Zero point correction (kcal/mol)	Electronic and Zero point correction (Hartree)	Gibbs free Energy (kcal/mol)	Electronic and Gibbs Free Energy (Hartree)	Number of Imaginary frequencies
-1362.024976	187.2476	-1361.73	152.0405	-1361.79807	0

Figure 4S. Sub(2-OMs) + 18-crown-6

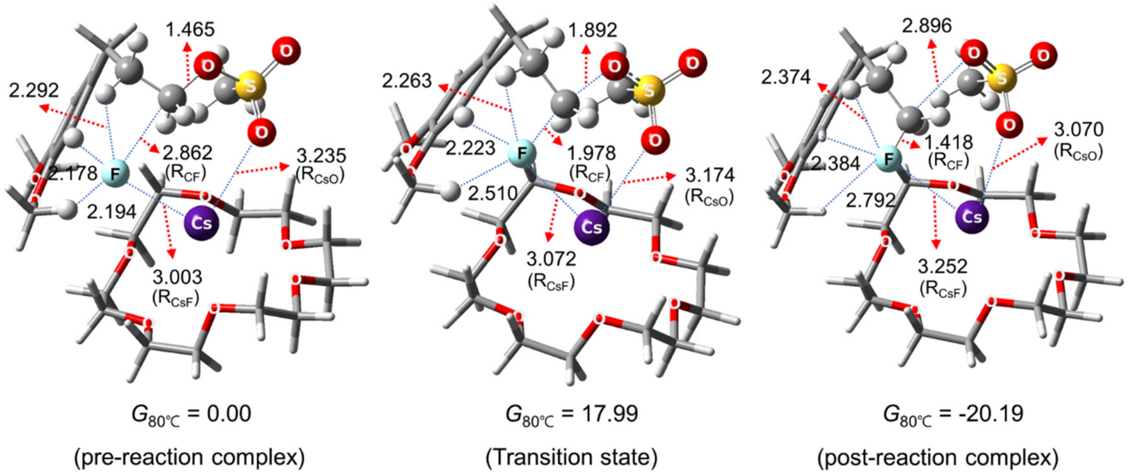

Sub(2-OMs) + 18-crown-6: pre-reaction complex

O	4.56809000	-2.51147300	1.51904300
O	2.98642900	-2.94209800	-0.48432000
C	5.33723600	-2.26788300	2.68554100
C	2.13676000	-3.20484500	-1.59610000
C	4.40840000	-1.47339200	0.65741300
C	3.55571900	-1.71467700	-0.44382300
C	4.98252000	-0.21980900	0.80460000
C	3.32458200	-0.71173400	-1.37757100
C	4.74312000	0.78280400	-0.14323600
C	3.93368700	0.54815800	-1.24552500
H	4.89584000	-1.46887100	3.28989400
H	6.36973700	-2.00754000	2.43370600
H	2.71945300	-3.21634000	-2.52461800
H	1.34745100	-2.45309100	-1.68185500
H	5.62087900	-0.00657100	1.65231300
H	2.59439200	-0.87458400	-2.16463500
H	5.21093000	1.75364900	-0.01360700
C	3.76670100	1.61870900	-2.30737200
H	3.96144400	2.59504100	-1.85256100
H	4.53995300	1.47603300	-3.07008000
C	2.40622600	1.64548400	-3.00480400
H	2.41987000	2.39304100	-3.80287000
H	2.15726600	0.68037000	-3.44881100
C	1.27032600	1.95019400	-2.06109800
H	0.30161500	1.85750000	-2.54689300
H	1.30456700	1.28981300	-1.19117600
F	0.49541100	-0.72278500	-2.72664300
Cs	-1.50277200	0.00599300	-0.60640000
H	1.72009800	-4.19664400	-1.42530300
H	5.32805100	-3.19742200	3.25151900
O	1.43035300	3.34048100	-1.62779300
S	0.69523000	3.78653400	-0.28485800
O	0.67631500	5.23311900	-0.31373300
O	-0.56698600	3.07321600	-0.17836200

C	1.82173500	3.22896100	0.95921800
H	1.39060500	3.47857600	1.92919900
H	1.93383600	2.15132500	0.86252600
H	2.76514500	3.75118600	0.80549200
O	-2.36664900	1.24123400	2.33134700
O	-2.24187200	-3.10835200	0.02862900
O	-4.43458200	0.77851600	0.38996000
O	0.35044000	0.61043700	1.78414900
O	-4.17008700	-1.44581300	-1.34859900
O	0.29809600	-2.06805500	0.87856800
C	-3.40233500	2.16022000	2.03934400
C	-1.26560000	1.88793100	2.94368400
C	-3.22491200	-3.56690800	-0.88250900
C	-1.00421600	-3.76631000	-0.18069100
C	-4.64675000	1.41618200	1.63378600
C	-0.12653200	0.91292500	3.07986600
C	-4.45639800	-2.70734100	-0.77198600
C	-0.04906400	-3.43548400	0.93812500
C	-5.57785200	0.06719800	-0.04251500
C	1.49558100	-0.22156400	1.75469300
C	-5.27382100	-0.55995700	-1.38547500
C	1.16600200	-1.68851800	1.92867400
H	-3.08642500	2.83811000	1.23446000
H	-3.63381000	2.76386400	2.92715100
H	-1.55158800	2.25591400	3.93834200
H	-0.94516500	2.73900700	2.32996200
H	-3.48386300	-4.61049300	-0.66068200
H	-2.84196600	-3.51765200	-1.91089100
H	-0.58550200	-3.45970500	-1.14767700
H	-1.15414000	-4.85426800	-0.20008700
H	-4.90072400	0.67324600	2.40188100
H	-5.47699100	2.13108900	1.55606900
H	0.67201600	1.37222400	3.67887000
H	-0.47071400	0.00786400	3.59531300
H	-5.28278500	-3.18823500	-1.31143900
H	-4.74020500	-2.61052200	0.28287300
H	-0.52384900	-3.66318400	1.90249800
H	0.85143900	-4.05673700	0.84121200
H	-5.85061500	-0.69336400	0.70109600
H	-6.43330000	0.74740000	-0.15671100
H	-6.16939800	-1.07488400	-1.75667300
H	-5.00689500	0.22596100	-2.09609000
H	0.68407700	-1.87259800	2.89902600
H	2.09420000	-2.27400600	1.88781900
H	1.94150800	-0.07555100	0.76710400
H	2.22055700	0.09017400	2.51931200

Electronic Energy (Hartree)	Zero point correction (kcal/mol)	Electronic and Zero point correction (Hartree)	Gibbs free Energy (kcal/mol)	Electronic and Gibbs Free Energy (Hartree)	Number of Imaginary frequencies
-2284.907998	423.302221	-2284.233423	359.7857094	-2284.334642	0

Sub(2-OMs) + 18-crown-6: transition state

O	4.55259700	-2.55728100	1.61458500
O	3.12374000	-2.99989000	-0.50175500
C	5.20200400	-2.30408300	2.84994200
C	2.28084000	-3.23342400	-1.62219000
C	4.42487400	-1.51127300	0.75797500
C	3.66212500	-1.75985900	-0.40450700
C	4.94414200	-0.24164100	0.96493000
C	3.47098300	-0.74884300	-1.33738500
C	4.72558700	0.77389400	0.02674800
C	4.00247100	0.53342200	-1.13331000
H	4.68385700	-1.52303500	3.41561600
H	6.24595000	-2.01375000	2.69794600
H	2.85516200	-3.19981200	-2.55414200
H	1.47652700	-2.49466400	-1.66159800
H	5.51258200	-0.02387100	1.85996100
H	2.82673000	-0.92242900	-2.18928800
H	5.13517900	1.76175100	0.21222400
C	3.82729000	1.63135400	-2.16282700
H	3.86328700	2.59687400	-1.65231600
H	4.67881800	1.61000000	-2.85173100
C	2.54774300	1.56173500	-2.99874800
H	2.52724300	2.41315100	-3.68208500
H	2.51854200	0.65799400	-3.60658200
C	1.28904600	1.57981500	-2.18267600
H	0.34681700	1.82453700	-2.63912700
H	1.29910800	1.21877300	-1.16563700
F	0.73418300	-0.27804700	-2.57617300
Cs	-1.58930300	0.09534700	-0.60197100
H	1.87274200	-4.23426800	-1.48977300
H	5.16550500	-3.23920900	3.40561900
O	1.49265000	3.38448700	-1.65393800
S	0.73786000	3.80372400	-0.39970300
O	0.63540400	5.26049800	-0.33254000
O	-0.53188200	3.07204000	-0.29152600
C	1.78801300	3.27222200	0.93379900
H	1.30194500	3.52511200	1.87606700
H	1.92115900	2.19411600	0.85815700
H	2.73987300	3.79443200	0.84214400
O	-2.35347600	1.17218200	2.39724500
O	-2.23663700	-3.08467600	-0.16956000
O	-4.49544500	0.67703600	0.54464500
O	0.33632300	0.57120100	1.71542200
O	-4.22642000	-1.38786000	-1.39700100
O	0.29417800	-2.09651100	0.74043700
C	-3.47794200	2.01412400	2.23571700
C	-1.23567600	1.89368100	2.88482900
C	-3.20926200	-3.51374300	-1.10504900
C	-1.01042100	-3.77046400	-0.35152300
C	-4.67752600	1.19007300	1.84992900
C	-0.06041300	0.96111200	3.01372200
C	-4.46629900	-2.70374400	-0.93158300

C	-0.07714900	-3.45790000	0.79001200
C	-5.62499300	-0.04591500	0.09570200
C	1.47634100	-0.26689000	1.67156000
C	-5.35462200	-0.53555300	-1.31046300
C	1.12716500	-1.73058800	1.82510900
H	-3.27985100	2.76971800	1.46275600
H	-3.69678000	2.53617600	3.17704200
H	-1.46599000	2.32438700	3.86878200
H	-0.98498100	2.70449000	2.18863800
H	-3.43960400	-4.57545300	-0.94581400
H	-2.83142700	-3.39355200	-2.12961900
H	-0.55938100	-3.47734600	-1.30855300
H	-1.18505200	-4.85463700	-0.37270300
H	-4.81009400	0.36857500	2.56664700
H	-5.57187400	1.82628600	1.88398100
H	0.76183100	1.48572300	3.51961800
H	-0.33927000	0.08967500	3.61938400
H	-5.27647000	-3.16492900	-1.51158300
H	-4.75335300	-2.70446000	0.12651400
H	-0.57957100	-3.67820500	1.74186100
H	0.81438700	-4.09540700	0.71534300
H	-5.83340600	-0.88017100	0.77806100
H	-6.51273200	0.60136500	0.07782200
H	-6.24902200	-1.04067400	-1.69750100
H	-5.13753800	0.32269700	-1.95093500
H	0.60533700	-1.91097400	2.77548900
H	2.04788100	-2.32950900	1.81656500
H	1.92551000	-0.11318700	0.68657000
H	2.20448000	0.02758400	2.43901800

Electronic Energy (Hartree)	Zero point correction (kcal/mol)	Electronic and Zero point correction (Hartree)	Gibbs free Energy (kcal/mol)	Electronic and Gibbs Free Energy (Hartree)	Number of Imaginary frequencies
-2284.879525	422.4017448	-2284.206385	359.9049362	-2284.30598	1

Sub(2-OMs) + 18-crown-6: post-reaction complex

O	4.70192100	-2.34174400	1.76800800
O	3.41876600	-2.99972100	-0.38261500
C	5.27952400	-1.97936400	3.01216200
C	2.61783400	-3.35680100	-1.49972400
C	4.52093800	-1.34832300	0.86073000
C	3.83126300	-1.71026700	-0.31667100
C	4.92332500	-0.03086300	1.02871000
C	3.59004800	-0.75580000	-1.29727800
C	4.63965000	0.92549300	0.04940100
C	3.97731700	0.58085200	-1.12163300
H	4.66839600	-1.23091400	3.52676000
H	6.29558300	-1.59467700	2.88350200
H	3.18761700	-3.28955300	-2.43183600
H	1.73491600	-2.71581900	-1.55828800

H	5.43985900	0.27026800	1.93110800
H	3.05368200	-1.04140400	-2.19134600
H	4.93678400	1.95622200	0.21560100
C	3.68645600	1.64120600	-2.16379100
H	3.46981500	2.58038700	-1.64640800
H	4.58793600	1.81546500	-2.76190300
C	2.52122900	1.34908900	-3.11181200
H	2.38749100	2.22181300	-3.75239400
H	2.75175200	0.50462300	-3.76851000
C	1.20509200	1.04613200	-2.40626800
H	0.38399800	1.65866900	-2.77342500
H	1.27472200	1.16555300	-1.32300500
F	0.83003300	-0.30263300	-2.63227000
Cs	-1.66084100	0.09219300	-0.57900300
H	2.31660000	-4.39061500	-1.33809300
H	5.31233500	-2.89078600	3.60588800
O	1.26961900	3.87988000	-1.81227000
S	0.53399700	3.91792400	-0.52901500
O	0.16406500	5.28215200	-0.10563000
O	-0.61012800	2.97592900	-0.50408900
C	1.69341000	3.32932100	0.69990300
H	1.20757600	3.33662500	1.67500700
H	2.00001500	2.31561700	0.44558200
H	2.55338700	3.99944300	0.70144800
O	-2.40560400	1.13840700	2.39305500
O	-2.06249100	-3.14109600	-0.19164900
O	-4.55544800	0.48468500	0.60291400
O	0.32888600	0.65759700	1.65714700
O	-4.18788800	-1.57756500	-1.34058000
O	0.43387700	-2.01929600	0.70330800
C	-3.58268700	1.90880500	2.24750900
C	-1.30571400	1.94649100	2.77921600
C	-3.00936900	-3.62825800	-1.12475000
C	-0.80272600	-3.76698800	-0.35949000
C	-4.73919300	1.00787700	1.90415100
C	-0.08305500	1.08119600	2.93972000
C	-4.32410500	-2.92327100	-0.92105200
C	0.10550000	-3.39156400	0.78260900
C	-5.62762200	-0.34912200	0.20733800
C	1.50622600	-0.12642900	1.64571400
C	-5.37610200	-0.81579400	-1.20974500
C	1.21270900	-1.60055100	1.81096200
H	-3.44977900	2.66217400	1.45850500
H	-3.80863900	2.43405800	3.18527600
H	-1.52545900	2.44662000	3.73197300
H	-1.11437700	2.70459100	2.00816400
H	-3.15429400	-4.70748700	-0.98368900
H	-2.65448400	-3.46056200	-2.15097500
H	-0.36164700	-3.46707600	-1.31946800
H	-0.92346100	-4.85852300	-0.36511600

H	-4.80596000	0.19167300	2.63586500
H	-5.66922800	1.59005800	1.94969100
H	0.71347300	1.66801200	3.41748200
H	-0.31470700	0.22461300	3.58605600
H	-5.09838800	-3.42556900	-1.51557300
H	-4.60697300	-2.98171400	0.13658700
H	-0.40319900	-3.60322800	1.73286500
H	1.01829900	-4.00097100	0.73910800
H	-5.71864300	-1.19777600	0.89788900
H	-6.57441500	0.20786700	0.23174300
H	-6.24201000	-1.39011500	-1.56308900
H	-5.25332600	0.05678200	-1.85597600
H	0.66271300	-1.78660100	2.74415500
H	2.15425600	-2.16561200	1.84675700
H	1.97251800	0.03434900	0.67024200
H	2.20299000	0.20758000	2.42542900

Electronic Energy (Hartree)	Zero point correction (kcal/mol)	Electronic and Zero point correction (Hartree)	Gibbs free Energy (kcal/mol)	Electronic and Gibbs Free Energy (Hartree)	Number of Imaginary frequencies
-2284.932196	422.8673569	-2284.258313	358.0926887	-2284.361538	0
